# Is Generalized Joint Hypermobility Associated with Chronic Painful Temporomandibular Disorders in Young Adults? A Cross-Sectional Study

**DOI:** 10.3390/jcm14010044

**Published:** 2024-12-25

**Authors:** Cláudia Barbosa, Sandra Gavinha, Tânia Soares, Tiago Reis, Conceição Manso

**Affiliations:** 1 FP-I3ID, Faculty of Health Sciences, University Fernando Pessoa, 4200-150 Porto, Portugal; sgavinha@ufp.edu.pt (S.G.); tsoares@ufp.edu.pt (T.S.); tiagofaria@ufp.edu.pt (T.R.); cmanso@ufp.edu.pt (C.M.); 2 RISE-Health, University Fernando Pessoa, 4200-150 Porto, Portugal; 3 CDRSP, Polytechnic Institute of Leiria, 2430-028 Marinha Grande, Portugal; 4 LAQV-REQUIMTE, University of Porto, 4051-401 Porto, Portugal

**Keywords:** musculoskeletal diseases, generalized joint hypermobility, orofacial pain, temporomandibular disorders

## Abstract

**Background/Objectives:** Generalized joint hypermobility (GJH) is a common condition characterized by an increased range of motion across multiple joints. Previous studies have suggested a possible association between GJH and temporomandibular disorders (TMDs). This study aimed to assess the prevalence of GJH in a Portuguese population of young university adults and to explore the relationship between GJH, temporomandibular joint (TMJ) symptoms/clinical findings, chronic painful TMDs, and chronic painful TMDs subtypes (myalgia, arthralgia, or combined myalgia and arthralgia). **Methods:** A cross-sectional study was carried out in Oporto university institutions, involving 1249 students (18–25 years). GJH was assessed using the Beighton score cut-off ≥ 4. TMJ symptoms and clinical findings were collected using the Research Diagnostic Criteria for TMD protocol, as well as TMD diagnoses. Univariate and multivariate analyses were carried out to examine the associations between GJH and the variables of interest. **Results:** The overall prevalence of GJH was 41.9%, with females exhibiting a significantly higher likelihood of GJH (*p* < 0.001). A statistically significant association was found between GJH and TMJ clicking (*p* < 0.05). Although no overall association was found between GJH and chronic painful TMDs, GJH was significantly associated with the combined diagnosis of myalgia and arthralgia (*p* < 0.05). **Conclusions**: The results suggest that GJH may be associated with the more complex subtypes of chronic painful TMDs. However, due to the small size effect of this association, future longitudinal studies with large samples using GJH broader diagnostic criteria are essential to elucidate the relationship between GJH and painful TMDs in asymptomatic nonsyndromic joint hypermobility populations.

## 1. Introduction

Joint hypermobility is not a diagnosis itself but rather a descriptor used to describe a joint that extends beyond its normal range of motion [1], and when it occurs across multiple joints, it is usually referred to as generalized joint hypermobility (GJH) or joint laxity [2]. GJH is common in the general population, and the prevalence tends to decrease with age and be more frequent in females and in the Asian and African populations than in Caucasians [3]. Asymptomatic nonsyndromic joint hypermobility, hypermobility spectrum disorders, and Ehlers–Danlos syndromes (especially the hypermobile type) are the most prevalent phenotypes in children and adults with GJH [4]. The Beighton Scoring System, a 9-point scale, is commonly utilized in research to assess articular hypermobility in adults and has been integrated into both current and past diagnostic criteria for hypermobility syndromes [5,6,7,8,9]. Despite its limitations [6,7], the system remains widely accepted, with a Beighton score of ≥4 frequently used as the threshold to define GJH in young adults at university [10,11,12].

Temporomandibular disorders (TMDs) are a heterogeneous group of conditions affecting the temporomandibular joints (TMJs), the jaw muscles, and the related structures associated with clinical signs and symptoms, such as functional limitation and joint sounds, muscle and/or joint pain [13,14]. A recent meta-analysis [15] reported a global incidence of TMDs of 34%, with individuals aged 18–60 years identified as the most affected. Geographic variations were noted, with North America (26%) and Europe (29%) exhibiting lower prevalence rates compared to Asia (33%) and South America (47%). Although TMDs represent one of the main causes of non-odontogenic pain in the orofacial region, it should be noted that being labeled as having TMD does not strictly imply that it is a painful condition. However, the most common subtypes of TMDs include pain-related disorders, such as myofascial pain and arthralgia, and disorders associated with the TMJ, primarily internal derangements and degenerative joint disease [13]. From a biopsychosocial perspective, the importance of preventing orofacial chronic pain conditions in young adults is recognized to avoid the detrimental effect of pain on overall well-being [16,17]. Although some studies [18,19,20,21,22] suggest an association between GJH and TMJ symptoms or articular TMDs, others have not supported this finding [23,24]. Population-based studies by Hirsch et al. and Chang et al. [18,19] examined painful TMDs but did not explore the relationship between GJH and painful TMD subtypes. Although studies have linked EDS with orofacial pain and a high prevalence of painful TMD subtypes [25,26] and chronic pain in general [27,28], to the author’s knowledge, no existing literature has explored the potential association between GJH and painful TMD subtypes in young adult populations. Therefore, the aims of the present study were as follows: (1) to determine the prevalence of GJH in a Portuguese university population of young adults (aged 18–25 years), according to the Beighton score cut-off ≥ 4; (2) to explore the associations between GJH and mechanic TMJ symptoms and clinical findings (clicking and mouth limitation opening), being the null hypothesis that there is no association between GJH and mechanic TMJ symptoms and clinical findings; and (3) to evaluate if GJH is associated to chronic painful TMDs and different chronic painful TMDs subtypes (myalgia, arthralgia, combined myalgia and arthralgia), being the null hypothesis that there is no association between GJH and chronic painful TMDs and different chronic painful TMDs subtypes.

## 2. Materials and Methods

The study protocol was initially approved by the Ethics Committee of the University Fernando Pessoa (UFP 26 November 2013) and subsequently by all institutions visited.

Potential participants were informed through institutional websites and emails. Interested students attended a session where they reviewed a flyer outlining each study stage and were informed of their right to withdraw at any point. After signing consent forms, the students completed self-report questionnaires, and clinical exams were conducted. No financial compensation was offered for participation.

### 2.1. Study Participants

A cross-sectional study was carried out from March 2014 to July 2014 in nineteen public and private university institutions from Oporto District (Portugal). The sample size of a minimum of 1022 was estimated using a power analysis technique [21] based on a population of 24,159 university students registered in those institutions during the school year of 2014, an expected TMD-symptom prevalence of 50% [29], a confidence level of 95%, and a precision of ±3%. Non-probabilistic sampling was used, which adjusts the number of subjects to be enrolled based on the proportion of students of the public (67%) versus private institutions (33%). Data collection began with public institutions, where 931 students voluntarily participated and completed all phases of the study. Following this, private institutions were visited, and data collection concluded once the target quota of 33% was reached, corresponding to 450 students who completed all phases. The final sample of 1381 university students reflected a distribution that did not significantly differ from the estimated distribution (Chi-Square Test, χ2 = 0.072, *p* = 0.789).

Given the broad age range of the student participants (18–67 years), for the purposes of the present study, only those aged 18 to 25 years were included. Additionally, students diagnosed with hypermobility syndromes, undergoing orthodontic treatment, those with lip lesions, and those who had undergone oral surgery (within the last month) or orthognathic surgery (within the last three months) were excluded, as were those with physical limitations that prevented proper seating in the observation chair. The final sample comprised 1249 students.

### 2.2. Data Collection

Since the Portuguese Diagnostic Criteria for Temporomandibular Disorders (DC/TMD) version was unavailable during data collection, the Portuguese RDC/TMD Axis I Personal History Questionnaire (PHQ) was used [30]. This questionnaire gathered demographic information and assessed pain location, pain history, and other TMD-related symptoms for TMD classification.

The clinical examination followed the RDC/TMD protocol and was conducted by a single examiner (C.B.) trained to the standards of the International RDC/TMD Consortium (now INfORM). The examiner was calibrated with an official reference standard examiner. The inter and intra-examiner agreement was equal to or greater than 90%, with a kappa of at least 0.8 for all clinical examination parameters, indicating at least strong agreement [31]. A finger algometer (Wagner^Ⓡ^ 0–5 lb, Greenwich, CT, USA) was used prior to temporomandibular joints and muscle palpation, as specified by DC/TMD protocol, to improve procedural reliability. Imaging was not part of the examination.

For the Beighton Score, a set of nine maneuvers was realized according to the Beighton criteria [32], and a total possible score was 9 points, including 1 point for each of (1) hyperextension of either fifth finger metacarpophalangeal (MCP) joint beyond 90° (measured only at the MCP, not including the interphalangeal joints); (2) apposition of either thumb to the ipsilateral forearm, so that the thumb touches the skin; (3) hyperextension of either elbow beyond 10°; (4) hyperextension of either knee beyond 10°; (5) forward flexion of the spine, so that the palms lay flat on the floor directly in front of the feet, while knees are fully extended. Thus, each joint and each side were scored independently, and small and large joint manual goniometers were used for any doubtful joint, and if any uncertainty remained about any of the maneuvers, the lower value of the Beighton score was recorded [33]. The examiner (C.B.) was trained and calibrated with an expert orthopedic physician. For calibration, ten young adults were accessed, and a Kappa of 0.88 was registered, indicating strong agreement. Subsequently, intra-examiner calibration was conducted, resulting in a perfect Kappa value of 1, indicating excellent agreement [31].

### 2.3. Data Analysis and Reduction

Categorical variables were expressed as percentages, and quantitative variables as mean and standard deviation (SD) and/or median and interquartile range (IQR) unless otherwise specified. There was no missing data for any variable. For all analyses reported, probability values are two-tailed, and the significance level was set at *p* < 0.05. IBM SPSS^®^ Statistics for Windows, Version 26.0 (IBM Corporation, Armonk, NY, USA) was used.

The students were divided into two groups according to their sex (male, female). The RDC/TMD Axis I diagnosis for this study included pain diagnoses (myalgia and arthralgia) and mechanical TMDs (disc displacements, arthritis, and osteoarthrosis) [26]. The first method categorized them based on pain severity: chronic painful TMDs (myalgia and/or arthralgia lasting at least six months), other TMDs (myalgia or arthralgia lasting less than six months, or any mechanical TMDs without pain), and TMD-free. The second focused on subtypes of chronic painful TMDs, dividing them into myalgia, arthralgia, and a combination of both. The Beighton Scoring System evaluates joint hypermobility on a scale from 0 to 9 points, and GJH was defined using the Beighton score cut-off of 4 points or higher [32].

Univariate associations between categorical variables were tested using the chi-square tests. Univariate logistic regression was used to predict the relationships between mechanic TMJ symptoms and clinical findings (clicking and mouth limitation opening) with GJH. To compare group differences, the effect size was quantified using an odds ratio (OR) with a 95% confidence interval (95% CI). OR values were interpreted as follows: 1.5 indicated a small effect size, 2 represented a medium effect size, and 3 reflected a large effect size [34]. Multivariable binary logistic regression models were used to test the association between the presence of GJH and chronic painful TMDs and between GJH and chronic painful TMD subtypes (myalgia, arthralgia, and combined myalgia and arthralgia). These models consisted of the Wald backward stepwise method, *p* = 0.05 for covariate inclusion and *p* = 0.10 for exclusion; for bias control, backward and forward stepwise methods were tested, and they converged, yielding the same results.

## 3. Results

In this study sample of 1249 young adults aged 18–25 years, 965 (77.3%) were females, with a mean age of 20.7 (SD ± 1.7) years and a median age (IQR) of 20 (19–22) years. Male students (median 21 (20–22) years) were slightly older than female students (median 20 (19–22) years; Mann–Whitney test, *p* < 0.001). Most students (99.1%) identified themselves and their ancestors as European/Caucasian, while 0.9% identified as African.

### 3.1. Prevalence of Generalized Joint Hypermobility

Two hundred and fifteen students (17.2%) had a null Beighton Score (n = 100 males; n = 115 females), and thirty-four female students (2.7%) had a Beighton Score of nine points. Beighton Score mean (±SD) was 3.2 (±2.4), and the median (IQR) was 3 (2–5) points. Female students (median 3 (2–5) points) have greater hypermobility scores than male students (median 2 (0–3) points; Mann–Whitney test, *p* < 0.001). The overall prevalence of GJH was 41.9%, with females exhibiting a significantly higher likelihood of GJH (48.2%) compared to males (20.4%) (OR = 3.62; Chi-square test, *p* < 0.001). No association was identified between GJH and ethnicity (Table 1).

### 3.2. Prevalence of Painful Temporomandibular Disorders and Temporomandibular Disorders Symptoms and Clinical Findings

The prevalence of any TMD was 38.4% (n = 480), with 27.2% (95%CI: 24.8–29.8%; n = 340) having chronic painful TMDs and 11.2% (95%CI: 9.5–13.1%; n = 140) other TMDs. Among students with chronic painful TMDs, 9.9% had myalgia, 8.3% had arthralgia, and 9.0% had combined myalgia and arthralgia. The prevalence of chronic painful TMDs in females was 29.6%, while in males was 19.0%. When assessing the relationship between painful TMDs and sex, it was determined that females were significantly more likely to exhibit chronic painful TMDs (Chi-square test, *p* < 0.001), with an estimated OR of 1.82. The prevalence of other TMDs in females was 11.1%, while in males was 11.6%. No association was identified between other TMDs and sex (Chi-square test, *p* > 0.05) (Table 2).

The prevalence of mechanic TMJ symptoms was 45.6% (n = 569) for clicking and 18.7% (n = 233) for mouth opening limitation. The prevalence of TMJ clinical findings was 41.5% (n = 518) for clicking and 0.7% for mouth opening limitation (<35 mm) (Table 3).

### 3.3. Associations Between Joint Hypermobility, Mechanical Temporomandibular Joint Symptoms/Clinical Findings and Chronic Painful Temporomandibular Disorders Diagnoses

Univariate analyses (Table 3 and Table 4) showed a significant association between GJH and the TMJ symptoms of clicking (OR = 1.27; *p* = 0.041) and mouth opening limitation (OR = 1.33; *p* = 0.048). They also presented a significant association between GJH and the TMJ clinical findings of clicking (OR = 1.28; *p* = 0.035) but not with mouth opening limitation (<35 mm) (*p* = 0.602) (Table 3).

Upon completion of the multiple regression analysis that included the variables GJH, ethnicity, age, and sex, the model retains GJH (OR = 1.25; *p* = 0.099), but only age (OR = 1.10; *p* = 0.015) and sex (OR = 1.93; *p* < 0.001) as independent variables associated with chronic painful TMDs (Table 4).

The prevalence of single diagnoses of myalgia and arthralgia and the combined diagnosis of myalgia and arthralgia was higher among young adults with GJH than in those without. Specifically, myalgia was observed in 15.4% of individuals with GJH compared to 12.9% without GJH, arthralgia in 13.7% versus 10.7%, and the combined diagnosis in 16.1% versus 10.4% (Table 5). Only the prevalence of the combined diagnosis of myalgia and arthralgia showed a statistically significant difference (Chi-square test, *p* < 0.013, OR = 1.65, 95% CI = 1.11–2.46). To determine the strength of the association between the chronic painful TMD subtypes and GJH, multivariate analyses adjusted for age and sex were employed. The results revealed that GJH was associated with the combined diagnosis of myalgia and arthralgia (OR = 1.43; *p* = 0.047) (Table 5).

## 4. Discussion

The present study assessed the prevalence of GJH in young adults using BS cut-off ≥ 4 and explored the relationship between GJH and mechanic TMJ symptoms and clinical findings, painful TMDs and painful TMD subtypes (myalgia, arthralgia, or combined myalgia and arthralgia).

GJH prevalence comparisons must be made carefully because it depends on the criterion used (cut-off for Beighton score), age, gender, and ethnicity of the sample assessed [7]. Studies on university young adults, using Beighton score ≥ 4, have reported GJH prevalence rates ranging from 26.8–57.1% [10,11,12]. The current study reported a GJH prevalence of 41.9% (48.2% female; 20.4% male), consistent with the study of Russek and Errico [10] of 40.1% (51.0% female; 27.4% male), among 267 American college and graduate students aged 17–26. In the present study, GJH was significantly more frequent in females (OR = 3.62, a large effect size) and consistent with the study of Russek and Errico [10] and other previous findings in university students [35,36]. However, gender differences in prevalence may be influenced by ethnicity. The study of Russek and Errico [10] included 81.3% North American Caucasians, alongside 9.7% Asians and 4.1% Africans, groups in which GJH tends to be more prevalent [3]. The present study’s sample was predominantly composed of European/Caucasians, and no association was observed between GJH and ethnicity. This demographic homogeneity may partly explain variations in gender GJH prevalence across the studies. Antonio and Magalhaes carried out a study involving 388 Brazilian university students aged between 18 and 25, reporting a prevalence of GJH of 26.8%, lower than that observed in the present study. On the other hand, Alghadir et al. examined 280 Egyptian university students in the same age group and found a higher prevalence of GJH of 57.1% [12]. It should be noted that none of the studies provided details on the ethnicity of their samples, which may have probably contributed to the differences observed in the prevalence rates compared to the present study. In relation to the study of Pasinato et al. that evaluated GJH and TMD, a prevalence of 54.1% was reported in a sample of 61 females aged 18 to 35 years [37]. This elevated prevalence may be attributable to the study’s exclusively female population, as GJH is more commonly observed in women [3] in the study by Hirsch et al. [18] conducted on a random German sample, the prevalence of GJH in the 20–29 age group (n = 212; 132 females) was reported as 16.0% (20.4% female; 8.8% male). In that study, a smaller sample size was examined, with a lower proportion of women and a broader age range. Additionally, the authors did not provide information about the sample’s ethnicity. The combination of factors could contribute to the differences observed compared to the present study.

Associations were found between GJH and mechanic TMJ symptoms (i.e., clicking, mouth opening limitation) and with the TMJ clinical findings of clicking but not with a mouth opening limitation (<35 mm). Similar to findings by Kulesa-Mrowiecka et al. [21], which demonstrated an association between GJH and joint clicking, the present study also shows that young adults with GJH are more likely to experience TMJ clicking. In women-based studies using magnetic resonance imaging [22,23], GJH was not a risk factor for joint disc displacement. However, more recent research on adolescents with GJH indicated a greater risk of developing TMJ disc displacement in those younger populations [20]. The more frequent clicking sounds in the GJH young adults are in accordance with the hypothesis that GJH predisposes the patients to joint instability [38] and could be related to jaw joint sounds and jerky movements [39]. However, these findings should be interpreted with clinical caution due to the very small effect size (OR < 1.5) observed. The results of the present study also indicated that young adults with GJH reported a greater perceived limitation in mouth opening. However, this perception did not correspond to a significant clinical finding that GJH was associated with a real mouth opening limitation, which is also aligned with Hirsch et al. study [18], corroborating the fact that reduction in proprioception associated with GJH can cause an increased range of movement without awareness [38,40]. Hence, the null hypothesis that there is no association between GJH and mechanic TMJ symptoms and clinical findings is partially rejected.

The present study adopted Axis I RDC/TMD in clinical evaluation, however, for the purpose of TMD classification, only the painful TMD diagnoses that are uniformly reliable and valid according to the revised RDC/TMD Axis I TMD diagnostic algorithms [41] were used to assess the associations with GJH. In the present study, the overall TMD prevalence was 38.4%, consistent with Zielinski et al. [15], who reported in Europe for the age group 18–60 years a TMD prevalence of 41%. In relation to chronic painful TMDs, the prevalence was 27.2%, comparable to the findings reported from a Swedish healthy dental students’ study (27.8%). As seen in the OPPERA study [42], both being a woman and the age increment was associated with a greater probability of having a chronic painful TMD.

In the present study, single chronic diagnoses of myalgia and arthralgia were more prevalent in young adults with GJH compared to those without. Notably, the combined chronic painful diagnosis of myalgia and arthralgia was significantly more frequent in individuals with GJH. Nonetheless, the odds ratio (OR = 1.65) indicates the likelihood of this combined diagnosis being higher in individuals with GJH compared to those without. While statistically significant (*p* < 0.013), the OR value suggests a modest association. These findings are not directly corroborated by any study that relates TMD with GJH in asymptomatic nonsyndromic joint hypermobility. However, Pasinato et al. (2015) observed that individuals with both TMDs and GJH had higher asymmetrical muscle activity, likely as a compensatory mechanism for joint stabilization due to weaker ligaments and possible proprioceptive issues [43]. These can lead to a TMJ overload and, in time, induce inflammatory joint changes [3,44], which could explain the appearance of more prevalent combined chronic painful TMDs (myalgia and arthralgia) in young adults with GJH.

Hirsch et al. [18] and Chang et al. [19] only found associations between GJH and disc-related TMDs, and no association was observed between GJH and painful TMDs. In those studies, the various painful TMD subtypes were analyzed without distinguishing between single or combined diagnoses of myalgia and arthralgia, a limitation previously highlighted by Dworkin and Huggins [45] relative to Hirsch et al.’s study [18]. Furthermore, the categorization of cases with concurrent disc displacements and painful diagnoses was not clearly defined in those studies. So, variations in TMD classification criteria across studies may, therefore, account for the observed differences in the present study.

Chronic musculoskeletal pain is one of the most challenging complications associated with GJH [27,28]. In EDS patients, the occurrence of painful TMDs has been widely studied. Giacomo et al. [25] reported elevated rates of myalgia (83%) and arthralgia (70%) in patients with EDS and TMDs, while Bech et al. [26] identified significantly higher occurrences of TMDs-related myalgia, arthralgia, and associated headaches in hypermobile EDS patients compared to controls. In the past, research on asymptomatic nonsyndromic GJH has prioritized mechanical TMJ dysfunction over painful TMDs. In the current study, no overall association was identified between GJH and chronic painful TMDs, and the null hypothesis that there is no association between GJH and chronic painful TMDs was accepted. However, an independent association was identified between GJH and the chronic combined diagnosis of chronic myalgia and arthralgia, rejecting the null hypothesis that there is no association between GJH and different chronic painful TMD subtypes. Nonetheless, the OR of 1.43 (*p* = 0.047) indicates a statistically significant but relatively modest association, suggesting a small effect size in clinical terms. These findings highlight the need for longitudinal studies, in large samples, investigating the relationship between asymptomatic nonsyndromic joint hypermobility and subtypes of painful TMDs to elucidate the role of GJH as a potential risk factor for chronic painful TMDs in young adults. Such research could offer valuable insights into the progression of TMDs in individuals with GJH, facilitating the development of targeted prevention and management strategies [4,16,17]. Additionally, it may help uncover the underlying pain mechanisms associated with these conditions [8].

A limitation of the present study is the potential selection bias, as the sample may not accurately represent the entire university student population in the Oporto district. Other potential bias stems from clinical examinations being performed by a single examiner. To mitigate this, the examiner underwent calibration for inter- and intra-examiner reliability, achieving a kappa coefficient of at least 0.8 across all clinical examination parameters. This value reflects strong agreement, supporting the consistency and reliability of the evaluations [31].

A major limitation of this study was the reliance on the Beighton score as the sole tool for assessing GJH. Critically, the Beighton score primarily evaluates upper limb joints, limiting its ability to directly identify GJH comprehensively [6,7]. Research highlights that the Beighton score, regardless of the cut-off used, should not be solely relied upon for diagnosing GJH, emphasizing the necessity of clinical judgment in its assessment [6,7]. Future studies should incorporate broader diagnostic criteria to better account for hypermobility spectrum disorders, excluding joint hypermobility syndromes, and to clarify the association between GJH and painful TMDs.

## 5. Conclusions

This cross-sectional study of young Portuguese university adults revealed a prevalence of GJH of 41.9% and suggests an association between GJH and chronic combined myalgia and arthralgia. However, due to the small size effect of this association, future longitudinal studies with large samples using GJH broader diagnostic criteria are essential to elucidate the relationship between GJH and painful TMDs in asymptomatic nonsyndromic populations.

## Figures and Tables

**Table 1:** Univariate analyses of Sex and Ethnicity in relation to Generalized Joint Hypermobility (GJH) (odds ratio (OR) and 95% confidence interval (95% CI)), according to Beighton Score (BS) cut-off ≥ 4.

	Gender		Ethnicity	
	Male	Female	European/Caucasian	African
GJH negative	226 (79.6)	500 (51.8)	721 (58.2)	5 (45.5)
GJH positive (BS ≥ 4)	58 (20.4)	465 (48.2)	517 (41.8)	6 (54.5)
*p*	<0.001		0.392	
OR (95% CI)	3.62 (2.65–4.97)		1.67 (0.51–5.51)	

All values reported are n (%) unless otherwise stated.

**Table 2:** Frequency and relative frequency of Temporomandibular Disorder (TMD)-free university students (n = 769) and with other TMDs (n = 140), as well as TMD-free university students (n = 769) and with painful TMDs (n = 340) by sex, their association, and evaluation of other TMDs and painful TMD odds for females (odds ratio (OR) and 95% confidence interval (95% CI)).

	TMDs-Free	Other TMDs	Painful TMDs	Other TMDs vs. TMD-Free		Painful TMDs vs. TMD-Free	
				OR (95% CI)	*p* Value	OR (95% CI)	*p* Value
Male	197 (69.4)	33 (11.6)	54 (19.0)	1	0.680	1	<0.001
Female	572 (59.3)	107 (11.1)	286 (29.6)	1.12 (0.73–1.70)		1.82 (1.31–2.54)	

All values reported are n (%) unless otherwise stated.

**Table 3:** Univariate analyses of mechanic temporomandibular joint (TMJ) symptoms and clinical findings (clicking and mouth opening limitation) in relation to Generalized Joint Hypermobility (GJH) (odds ratio (OR) and 95% confidence interval (95% CI)), according to Beighton Score (BS) cut-off ≥ 4.

	TMJ Symptoms				TMJ Clinical Findings			
	Clicking		Mouth Opening Limitation		Clicking		Mouth Opening Limitation (<35 mm)	
	No	Yes	No	Yes	No	Yes	No	Yes
GJH negative	413 (56.9)	313 (43.1)	604 (83.2)	122 (16.8)	443 (61.0)	283 (39.0)	720 (99.2)	6 (0.8)
GJH positive (BS ≥ 4)	267 (48.7)	256 (48.9)	412 (78.8)	111 (21.2)	288 (55.1)	235 (44.9)	520 (99.4)	3 (0.6)
*p*	0.041		0.048		0.035		0.602	
OR (95% CI)	1.27 (1.00–1.58)		1.33 (1.00–1.78)		1.28 (1.02–1.60)		0.69 (0.17–2.78)	

All values reported are n (%) unless otherwise stated.

**Table 4:** Multivariate * Analyses of risk factors independently associated with chronic painful Temporomandibular Disorders (TMDs) (odds ratio (OR) and 95% confidence interval (95% CI)).

	OR (95% CI)	*p* Value
GJH positive (BS ≥ 4)	1.25 (0.96–1.63)	0.099
Ethnicity	0.53 (0.31–7.54)	0.603
Age (1-year increment)	1.10 (1.02–1.18)	0.015
Sex	1.81 (1.28–2.56)	<0.001

* Generalized Joint Hypermobility (GJH) negative was the reference (Beighton Score (BS) < 4), Ethnicity (European/Caucasian was the reference), Sex (male was the reference), and Age (1-year increment).

**Table 5:** Multivariate Analyses of chronic painful Temporomandibular Disorder (TMD) subtypes associated with Generalized Joint Hypermobility (GJH) (odds ratio (OR) and 95% confidence interval (95% CI)), according to Beighton Score (BS) cut-off ≥ 4, with adjustment for age and sex.

	Painful TMDs					
	Myalgia		Arthralgia		Myalgia and Arthralgia	
	No	Yes	No	Yes	No	Yes
GJH negative	466 (87.1)	69 (12.9)	466 (89.3)	56 (10.7)	466 (89.6)	54 (10.4)
GJH positive (BS ≥ 4)	303 (84.6)	55 (15.4)	303 (86.3)	48 (13.7)	303 (83.9)	58 (16.1)
*p* value	0.538		0.388		0.047	
OR (95% CI)	1.13 (0.76–1.67)		1.21 (0.79–1.84)		1.43 (1.06–2.26)	

All values reported are n (%) unless otherwise stated.

## Data Availability

The data that support the findings of this study are available from the corresponding author upon reasonable request.
